# The Lived Experience of Participants in Preclinical Alzheimer’s Intervention Research

**DOI:** 10.1002/alz.085976

**Published:** 2025-01-09

**Authors:** Nancy F Meserve

**Affiliations:** ^1^ NA, Charlottesville, VA USA

## Abstract

Preclinical Alzheimer’s prevention trials require a multi‐year commitment from diverse, cognitively unimpaired individuals willing to receive biomarker results of confirmed Alzheimer’s pathology and possible ApoE4 status. Participants learn new terms such as ARIA, edema and microhemorrhage and undergo numerous MRI scans for safety monitoring. They take quarterly composite Alzheimer’s assessments that are anxiety‐provoking and highlight weaknesses which may have been unrecognized in daily life. Infusions are often described as an hour in length, yet study visits may require several hours biweekly for record‐updating, infusion preparation and delivery, monitoring, and travel. Such a commitment has few, if any, corollaries in prevention of asymptomatic disease. Researchers and clinical site staff can benefit from the lived experience of preclinical trial participants. Their individual priorities, risk/benefit calculations, preferences for information and disclosure, engagement with their communities and hope for themselves and others may differ significantly from those with confirmed diagnoses in trials of disease‐modifying treatments.

In this talk, a woman with two copies of ApoE4, in her third year of the AHEAD‐45 preclinical‐AD trial on lecanemab, will portray her experience of trial participation, She reports also on her conversations with other current and future participants in this or similar studies. The perspective of a participant on biomarker and genetic disclosure, risk discussion and safety monitoring, symptom reporting, unscheduled MRIs, disclosure of isolated ARIA‐H and resumption of infusions will extend the understanding of how to overcome barriers to participation by others. The acknowledgement of uncertainties and encouragement of dialogue and questions empowers participants to partner with researchers. Recognition of the views of family members more skeptical of prevention than treatment of diagnosed dementia or other life‐threatening diseases may only be possible by listening to participants' experiences. The importance of participation may be best received when shared personally, in community groups and online by participants whose lived experience perspectives speak to the hopes as well as the fears of those with high risk of Alzheimer’s dementia.

